# Microcystin-LR, a Cyanobacterial Toxin, Induces DNA Strand Breaks Correlated with Changes in Specific Nuclease and Protease Activities in White Mustard (*Sinapis alba*) Seedlings

**DOI:** 10.3390/plants10102045

**Published:** 2021-09-28

**Authors:** Márta M-Hamvas, Gábor Vasas, Dániel Beyer, Eszter Nagylaki, Csaba Máthé

**Affiliations:** Department of Botany, Faculty of Science and Technology, University of Debrecen, Egyetem tér 1, H-4032 Debrecen, Hungary; hamvas.marta@science.unideb.hu (M.M.-H.); vasas.gabor@science.unideb.hu (G.V.); beyerdani@gmail.com (D.B.); esztern@gmail.com (E.N.)

**Keywords:** microcystin-LR, *Sinapis alba*, programmed cell death, TUNEL, SSP nuclease, protease, in-gel activity assay

## Abstract

There is increasing evidence for the induction of programmed cell death (PCD) in vascular plants by the cyanobacterial toxin microcystin-LR (MC-LR). Our aim was to detect the occurrence of PCD-related DNA strand breaks and their possible connections to specific nuclease and protease activities. DNA breaks were studied by the deoxynucleotidyl transferase mediated dUTP nick end labeling (TUNEL) method in the photoperiodically grown dicot model of white mustard (*Sinapis alba*). In-gel nuclease and protease activity assays showed changes in the activities of specific isoenzymes during treatments with MC-LR. Strand breaks occurred both in the developing root epidermis and cortex. Several isoenzyme activities were related to these breaks, for example: an increase in the activity of neutral 80–75 kDa, acidic high MW (100–120 kDa) and, most importantly, an increase in the activity of neutral 26–20 kDa nucleases, all of them having single-stranded DNA cleaving (SSP nuclease) activities. Increases in the activities of alkaline proteases in the 61–41 kDa range were also detected and proved to be in relation with MC-LR-induced PCD. This is one of the first pieces of evidence on the correlation of PCD-related DNA strand breaks with specific hydrolase activities in a model dicot treated with a cyanobacterial toxin known to have environmental importance.

## 1. Introduction

Microcystin-LR (MC-LR) belongs to a family of toxic heptapeptides produced by several cyanobacterial genera [1]. The producers require relatively warm water, and global warming together with freshwater eutrophication induces their overproduction [1,2,3]. The release of MCs into water bodies at high concentrations affects not only aquatic ecosystems but also human and animal health [2]. Concerning vascular plants, it affects not only aquatic macrophytes naturally co-existing with toxic cyanobacteria but terrestrial crops (via spray irrigation with contaminated waters) as well [3,4]. The biochemical targets of MC-LR are mainly type 1 and 2A protein phosphatases [5]. Meanwhile, toxin exposures in the long-term and/or their relatively high concentrations induce a wide variety of stress responses, one of the most important being the induction of cell death [4]. Therefore, cellular/biochemical studies of MC-LR toxicity are important not only for using the toxin as a tool for a better understanding of protein phosphatase functioning but also for understanding its harmful environmental effects.

MC-LR is known to induce programmed cell death (PCD) and necrosis in many eukaryotes [6]. Concerning vascular plants, the particular effects of cyanotoxins may vary among species, but there are cell death symptoms detected in nearly all species studied. The most important are: nuclear condensation and fragmentation, the formation of micronuclei, DNA degradation/strand breaks related to nuclease activities and typical symptoms of necrosis [4,7,8,9]. However, there is still a need for further research to achieve a better understanding of MC-LR-plant interactions in this context. Therefore, we aimed to study the occurrence of DNA strand breaks in relation to nuclease and protease activities in a toxin-sensitive plant model for white mustard (*Sinapis alba*). For this, we applied terminal deoxynucleotidyl transferase mediated dUTP nick end labeling (TUNEL), a suitable method to visualize strand breaks at the tissue level [10,11] in concert with in-gel assays of single-strand preferring (SSP) nuclease and protease activities. These latter assays give us an insight not only in overall changes in enzyme activities in the presence of MC-LR but also the relevant changes in their specific isoenzymes as well [8,12]. We show here the occurrence of DNA breaks in roots and that specific nucleases/proteases are related to this PCD event. The present work is one of the first pieces of evidence for MC-LR-induced DNA strand breaks in relation to specific nuclease and protease activities in a model dicot plant.

## 2. Results

### 2.1. MC-LR Induces DNA Strand Breaks in Mustard Root Cells as Revealed by the TUNEL Reaction

**Figure 1:**
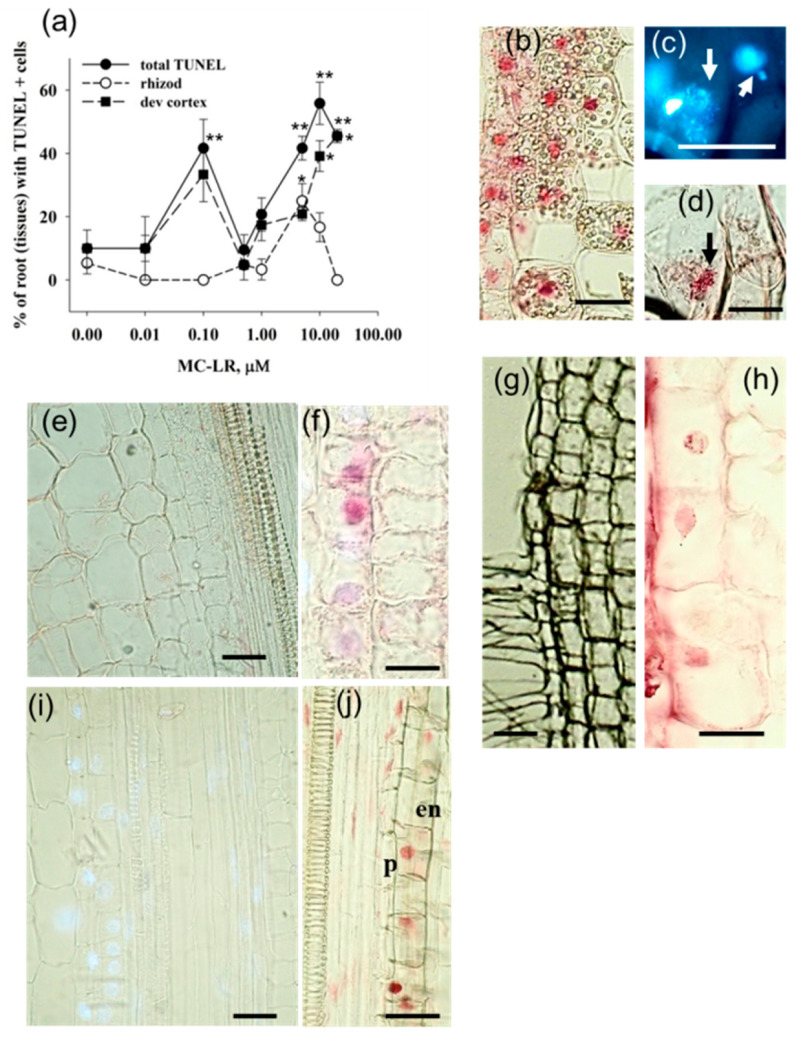
The effect of MC-LR on the induction of programmed cell death (PCD) in mustard roots exposed for 4 days to the cyanotoxin, as revealed by the TUNEL reaction in longitudinal sections. (**a**) The dose-dependent effects of MC-LR on the formation of TUNEL-positive cells and tissues (rhizod-rhizodermis/root epidermis; dev cortex: developing cortex tissue in the elongation and root hair zone). Significance of differences between controls and treated ones were evaluated by *t*-tests, Sigma-Plot 12.0; * *p* < 0.05; ** *p* < 0.001. (**b**–**d**): Root cortex cells from a root exposed to 20 µM MC-LR: (**b**) TUNEL reaction; (**c**) Different stages of nucleus fragmentation (arrows); (**d**) TUNEL reaction of the highly fragmented nucleus (arrow). (**e**) General view of developing cortex and stele of a control root. (**f**) TUNEL-positive developing root cortex cells in a root treated with 1 µM MC-LR. (**g**) Epidermis and outer cortex layers of a control root. (**h**) TUNEL-positive cells in the epidermis of a root treated with 5 µM MC-LR. (**i**) Developing inner cortex and stele of a control root. (**j**) TUNEL-positive cells in the endodermis (en) and pericycle (p) of a root treated with 20 µM MC-LR. Scalebars: 50 µm.

We have performed the TUNEL reaction in longitudinal sections of *S. alba* primary roots exposed to MC-LR for 4 days while we concentrated on differentiated regions of young root segments. During the analysis of the percentage of roots showing TUNEL positive cells, we found that for the overall root zones analyzed, MC-LR increased TUNEL positivity in a dose-dependent manner. There were maximal values at two distinct MC-LR concentrations: 0.1 and 10 μM (Figure 1a). These two maxima coincided with the two TUNEL positivity peaks in the developing root cortex (Figure 1a,e,f), while for root epidermis, a significant increase in TUNEL positivity was observed only at higher, 5–10 μM MC-LR concentrations (Figure 1a,g,h). High toxin concentrations increased TUNEL positivity in inner cell layers of roots: the endodermis and pericycle (Figure 1i,j). These layers are important in lateral root development, and, indeed, high MC-LR concentrations decreased the numbers of emergent laterals [13].

Combined TUNEL labeling and DAPI staining showed that TUNEL positivity was detectable not only in apparently intact but also highly fragmented nuclei as well (Figure 1b–d).

### 2.2. MC-LR Induces Changes in Single-Stranded DNase Activities in Mustard Seedlings

**Figure 2:**
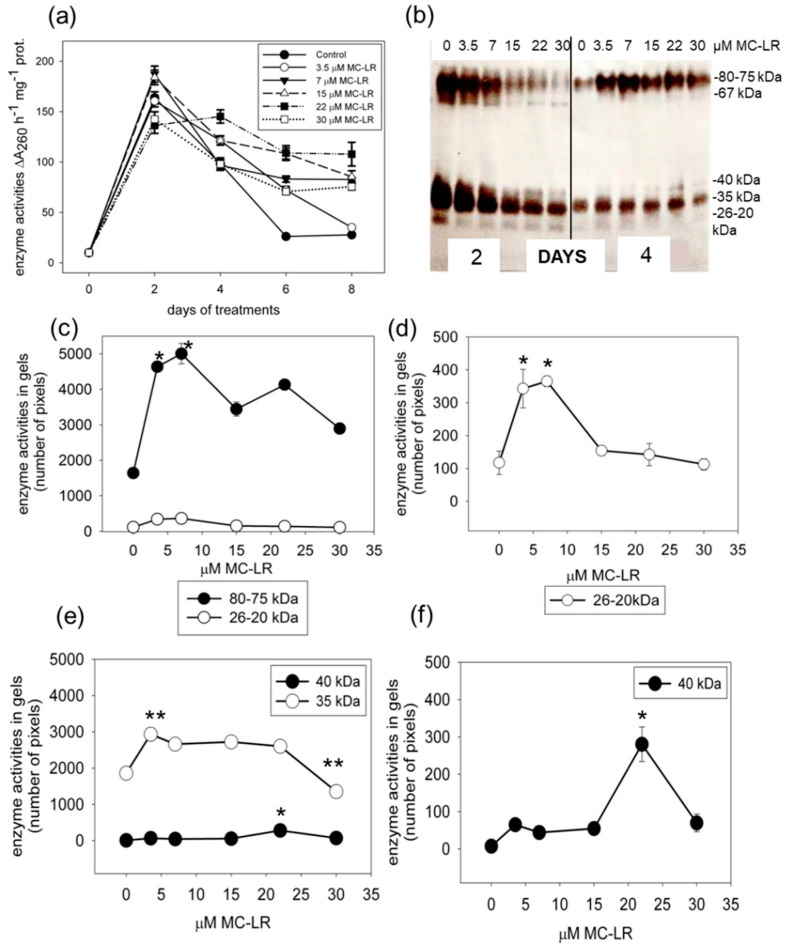
The time- and dose-dependent effects of MC-LR on neutral (assayed at pH 6.8) single-stranded DNase (SSP nuclease) activities of whole *S. alba* seedlings. (**a**) Time- and toxin-dose-dependent total SSP nuclease activities according to the spectrophotometric assay. (**b**) The in-gel activity assay shows characteristic MC-LR dependent changes in the 80–75, 40–35 and 26–20 kDa isoenzymes at 2 and 4 days of toxin treatments. (**c**) Quantification of activity gels by the CP Atlas method for the activities of the 80–75 and 26–20 kDa isoenzymes shows a clear MC-LR-induced increase for the 80–75 kDa isoenzymes at 4 days of exposure. (**d**) Shows a scale-up chart for the 26–20 kDa isoenzyme to see more clearly the increases induced by MC-LR at 4 days of exposure. (**e**,**f**) Quantification of the activities of the 40 kDa and 35 kDa isoenzymes. Elevated 40 kDa activity in 3.5–22 μM MC-LR-treated seedlings and an increase in 35 kDa activity at 4 days of 22 μM MC-LR treatment shown together in same scaling. (**f**) Shows a scale-up chart for the 40 kDa isoenzyme to see more clearly the increase induced by 22 μM MC-LR. Whole plant extracts containing 15–15 μg protein were loaded on 7.5–12.5% gradient gels. Gels were incubated for 17 h/28 °C. The significance of differences between controls’ and treated ones’ enzyme activities were evaluated after the analysis of variance using One-Way ANOVA by Tukey/Holm–Sidak method or *t*-test, Sigma-Plot; **: significance at *p* < 0.001; *: significance at *p* < 0.05.

The analysis of time- and MC-LR-concentration-dependent total neutral SSP nuclease activities (assayed at pH 6.8) showed two important results. Firstly, controls showed strongly time-dependent changes with high activities of two-day-old pre-germinated seedlings that decreased until eight days. This could be in relation to a general increase in hydrolase activities to provide low-molecular weight compounds for seedling nutrition (Figure 2a). Secondly, four days of exposure was the transition point for the effects of MC-LR, because starting from this treatment time, the toxin increased nuclease activities. The highest increase occurred at eight days of exposure, where controls exhibited low activities, while seedlings treated with the cyanotoxin showed high activities (Figure 2a). The in-gel analysis of the activities of SSP nuclease isoenzymes further confirmed this transition point. For controls, all isoenzymes showed relatively high activities, while MC-LR decreased these SSP nuclease activities after two days (Figure 2b). At four days of exposure, activities of all isoenzymes decreased for controls, while MC-LR increased their activities. At this time point, quantification of in-gel activities showed prominent and significant increases for the 80–75 kDa, 40 kDa, 35 kDa and 26–20 kDa isoenzymes (Figure 2c–f). Even the lowest (3.5 μM) examined toxin concentration caused a significant (*p* < 0.001, *t*-test) increase in the activity of an enzyme of 35 kDa. Its higher activity than the control was characteristic in seedlings treated with 7–22 μM MC-LR as well (Figure 2e).

**Figure 3:**
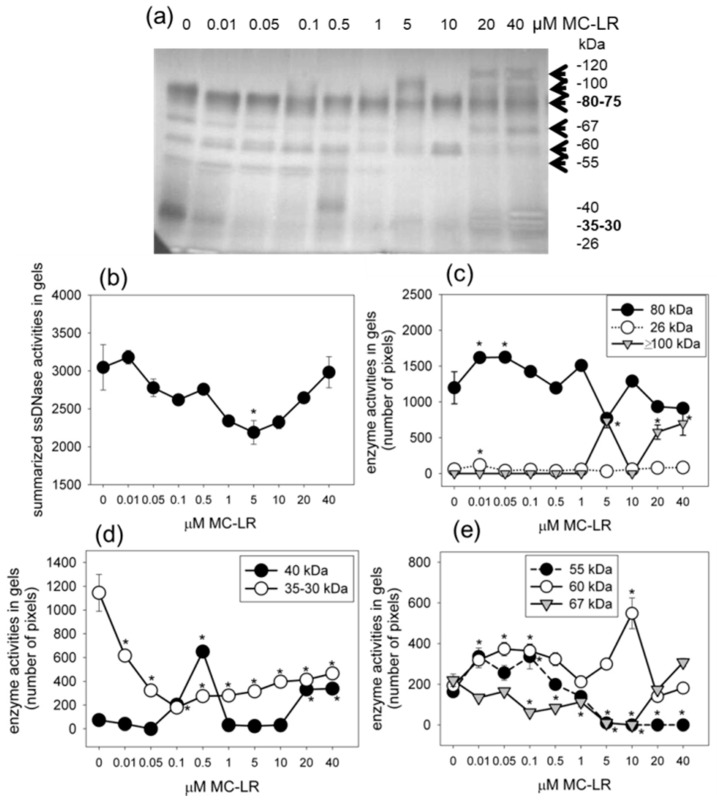
The dose-dependent effects of MC-LR on acidic (assayed at pH 5.5) single-stranded DNase (SSP nuclease) activities of whole *S. alba* seedlings after 4 days of exposure. (**a**) The in-gel activity assay shows characteristic MC-LR-dependent changes of the 120–100, 80–75, 67, 60 and 55 kDa isoenzymes (arrows). Gel was incubated for 6 h at 39 °C. (**b**) Quantification by the CP Atlas method based on activity gels of total acidic SSP nucleases shows their transient activity decrease. (**c**–**e**) MC-LR concentration-dependent changes in the activities of the ≥100 kDa, 80 kDa, 67 kDa, 60 kDa, 55 kDa, 40 kDa, 35 kDa and 26 kDa acidic SSP nuclease isoenzymes. * Indicates the activity differences of MC-LR treatments vs. controls at *p* < 0.05 significance.

Because of the characteristic alterations at the fourth day of MC-LR treatments in *S. alba* seedling tests, in the further experiments, we modified our test system and used only four-day-long treatments [14]. In addition, we used MC-LR at a wider concentration range. Concerning the acidic nuclease isoenzymes (assayed at pH 5.5) characteristic for 4 days of toxin exposure, we detected important differences as compared to the neutral SSP nucleases. Firstly, the activities of the characteristic neutral 80–75, 40–35 and 26–20 kDa nucleases decreased or almost completely disappeared here in controls (Figure 3a). Secondly, MC-LR did not markedly change the activities of these isoenzymes. For those of 80–75 kDa, there were slight increases at 0.01–0.05, 1 and 10 μM MC-LR, but these increases were significant only in the 0.01–0.05 μM MC-LR-treated plants (Figure 3c). For the 40 kDa enzyme, a double peak of increased enzyme activity was detectable. The peaks were at 0.5 and 20–40 μM MC-LR. Below their optimal pH, MC-LR decreased and did not increase the activity of the 35 kDa enzyme (Figure 3a,d; compare with Figure 2b,e). Thirdly, isoenzymes not detectable at pH 6.8 had activities at pH 5.5, and most of them were modulated by MC-LR; e.g., the activity of a 67 kDa isoenzyme was significantly decreased at low MC-LR concentrations and increased at high (20–40 μM) toxin concentrations (to 140% of controls, but this was statistically non-significant) (Figure 3e). A 60 kDa isoenzyme had significantly increased activity (as compared to controls) at both lower (0.05–0.1 μM) and at higher (10 μM) MC-LR concentrations, while the activity of a 55 kDa isoenzyme increased only at low concentrations (0.01 and 0.1 μM), but decreased at 5 μM and higher MC-LR concentrations (Figure 3a,e). Perhaps the most interesting isoenzymes were those of 120–100 kDa (large enzymes and/or enzyme complexes), because their activities were detectable only in the presence of MC-LR, while in controls, their activities were below the detectable levels (Figure 3a,c). In spite of these changes in the activities of particular acidic isoenzymes, the overall in-gel detectable activities showed a MC-LR concentration-dependent transient decrease. This decrease was significant at 5 μM MC-LR; then, activity re-increased to the levels of controls at higher MC-LR concentrations (Figure 3b).

### 2.3. MC-LR Induces Changes in Protease Activities in Mustard Seedlings

**Figure 4:**
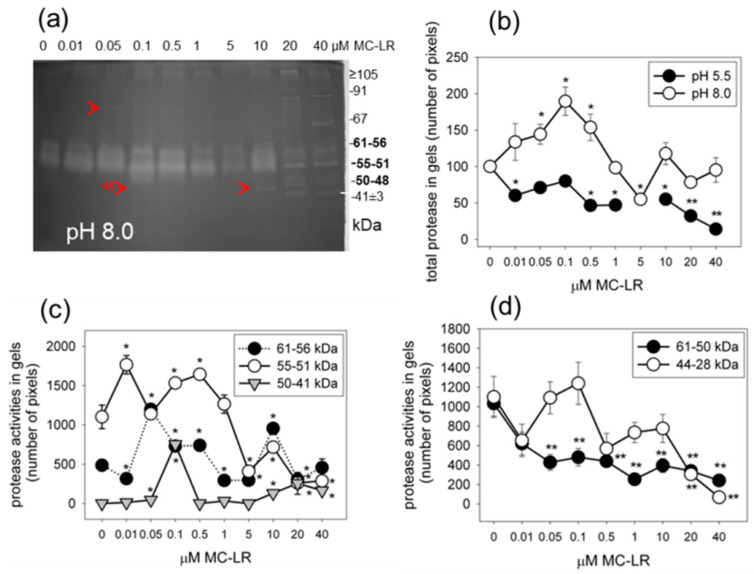
The dose-dependent effects of MC-LR on protease activities of whole *S. alba* seedlings after 4 days of exposure. (**a**) In-gel activity assay of alkalic proteases. Red arrowheads indicate isoenzymes with minor activities, but characteristic only for MC-LR treatments. (**b**) Quantification by the CP Atlas method based on activity gels of total acidic and alkaline protease activities, shown as percentages of controls (100%). (**c**) Quantification by the CP Atlas method based on activity gels of 61–56, 55–51 and 50–41 kDa protease activities under alkaline conditions. (**d**) Quantification by the CP Atlas method based on activity gels of 61–50 and 44–28 kDa acidic protease activities. (**b**–**d**) Shows that only the alkaline proteases show significant increases in the presence of MC-LR. Significance of differences between controls and treated ones were evaluated by *t*-tests, Sigma-Plot 12.0; * *p* < 0.05; ** *p* < 0.001.

The in-gel activity assay for proteases of 4-day treated seedlings showed that the most active proteases were isoenzymes in the 61–51 kDa range under alkaline (pH 8.0) conditions (Figure 4a,c). Under these conditions, MC-LR induced a transient and significant increase in the total protease activity (Figure 4b). That is, low toxin concentrations (0.01–0.5 μM) were responsible for this increase, while higher concentrations decreased protease activities back to the level of controls (Figure 4b). Changes in the activities of the 55–51 kDa enzymes followed this pattern for the effects of MC-LR, while the 61–56 kDa and 50–41 kDa isoenzymes showed a second peak of activity increase at treatments with 10 and 20 μM MC-LR as well (Figure 4a–c). Several isoenzymes with minor activities appeared only for seedlings treated with MC-LR, being undetectable in controls. Their MWs were in the ≥67 kDa and 50–41 kDa ranges (Figure 4a, red arrowheads and 4c for the 50–41 kDa isoenzymes).

Total acidic protease activities were decreased by MC-LR, and this was mostly true for the characteristic 61–50 kDa and 44–28 kDa isoenzymes (Figure 4b,d).

## 3. Discussion

Throughout our study we concentrated on 4 days of MC-LR treatments, because this time frame is enough to induce most of the PCD symptoms in mustard seedlings (time-course results shown for neutral nucleases in this study; [4]).

The TUNEL reaction indicates the presence of single- and/or double-stranded DNA breaks that release free 3′-OH ends [11,15]. The combined DAPI staining and TUNEL labeling in MC-LR-treated mustard roots showed TUNEL positivity but otherwise intact-looking nuclei, and the TUNEL reaction occurred in nuclei exposing extensive fragmentation as well (Figure 1). Strong chromatin fragmentation is characteristic for later stages of PCD [9]. Thus, the TUNEL procedure allowed us to detect several stages of plant cell death. The occurrence of DNA strand breaks was MC-LR dose-dependent showing two peaks of increase at 0.1 and 10 μM MC-LR and a slight decrease at the highest (20 μM) toxin dose investigated (Figure 1a). At such a high cyanotoxin dose, the onset of necrotic cell death occurred; thus, the complete disappearance of nuclear DNA can be seen in 4-day-old mustard seedlings [9,13,16]. Such transient changes in TUNEL positivity can be observed in roots of the gramineous aquatic plant *Phragmites australis* (reed grass) as well (data not shown). Revealing the causes for these two peaks of maximal TUNEL positivity needs further research, but, as we will see below, they are related to toxin-induced changes in nuclease and protease activities. Cylindrospermopsin (CYN), a protein synthesis inhibitory cyanotoxin, also increases the occurrence of TUNEL-positive nuclei both in mustard and reed grass [14]. We can state that both cyanotoxins have genotoxic effects in plants, as for all eukaryotes (shown for mammals by [6,17]).

Essentially, all endonucleases produce 3′-OH termini of cleavage products also detected by TUNEL [18]. Our hypothesis was formulated as follows. Can the induction of DNA strand breaks observed by the TUNEL assay be related to biochemical events that induce PCD? To verify this hypothesis, we investigated the nuclease (and in addition, protease) activities potentially related to DNA rupture in MC-LR-treated mustard seedlings.

The most important nuclease in relation to MC-LR-induced PCD seems to be the 80–75 kDa isoenzyme. MC-LR increased its activity, but this effect was dominant (about 305% of control) only in neutral conditions and in at least 4-day treatments (Figure 2 and Figure 3). In short-term (2 days) MC-LR exposures, where most of cell death symptoms are not detectable yet, the 80–75 kDa nuclease has high activity in controls that is decreased in the presence of MC-LR under neutral conditions (Figure 2). This isoenzyme has both SSP nuclease and double-stranded DNA cleaving (dsDNase) activity at least under acidic conditions [14].

Another group of isoenzymes are the transiently active 40–35 kDa nucleases with well visible activities at 2- and 4-day-old seedlings under neutral pH conditions (Figure 2e,f). They show increases in activity at 4 days of MC-LR exposure, while, again, at 2 days, MC-LR inhibits them. This isoenzyme group has no dsDNase activity [14]. Interestingly, their activities are increased more dramatically in dark-grown mustard seedlings (Figure S1). This makes it possible that one of them is related to an Arabidopsis neutral-alkaline nuclease inducible under dark conditions [19]. However, these isoenzymes show activities at acidic pH as well in white mustard (Figure 3).

The 26–20 kDa isoenzymes have low activities in controls at neutral and acidic pH. At 4 days of MC-LR exposure, they have increased, yet still low, activities as compared to controls (Figure 2c,d). These isoenzymes are dependent on bivalent cations [14]. A similar, Ca^2+^-dependent neutral 28 kDa isoenzyme was associated with DNA laddering during the treatment of oat leaves with fungal elicitors [20]. Thus, in spite of its relatively low activity, this isoenzyme might be associated with DNA strand breaks in TUNEL-positive mustard cells. However, this isoenzyme disappears almost completely after 6–8 days from the onset of experiments, and this is valid for controls as well. We can therefore suppose that the 26–20 kDa isoenzyme group is active only at the initial stages of PCD induction in toxin-treated (4 days of exposure) mustard seedlings.

Under acidic conditions, there is a group of high MW (120–100 kDa nucleases) that are active only in the presence of high concentrations of MC-LR; that is, they are not detectable in controls (Figure 3). The occurrence of these nuclease activities coincided with the second TUNEL-positivity peak (Compare Figure 1a and Figure 3a). Thus, the two TUNEL-positivity peaks (Figure 1a) might be attributed to activities of different nuclease isoenzymes. The 120–100 kDa large nucleases are rarely detected in plants. We can only speculate that they could be multimeric enzyme complexes, since several plant nucleases tend to form multi-subunit structures [21]. Another interesting nuclease group is of the 67–55 kDa MW. They are active under acidic conditions in the presence of Mg^2+^ ions (ion dependence is not shown here), and their overall activity increases at very high (10–40 µM) MC-LR concentrations (Figure 3a,e). This indicates that the 67–60 kDa nucleases are inducible mainly when tissue necrosis (typical for these high toxin concentrations) occurs. Meanwhile, the 60 kDa and 40 kDa acidic nucleases show increased activities at low MC-LR concentrations as well, showing the characteristic two peaks that can be related to the increase in TUNEL positivity (compare Figure 1 and Figure 3).

A comparison of the neutral SSP nuclease pattern of *S. alba* with that of *P. australis* (a previous study, [8]) shows fundamental differences. For both plants, isoenzyme activity patterns depend largely on developmental stage, but those of high molecular weight isoenzymes (≥67 kDa) in controls shown in this study are missing or very weak in *P. australis*. Meanwhile, the neutral isoenzymes of the 40–20 kDa range are present in both species, and their activities change in a similar manner with MC-LR treatments.

The 61–41 kDa proteases, characteristic in mustard seedlings, showed transient increases in their activities in the presence of MC-LR only under alkaline conditions (Figure 4). The isoenzymes fitting into the 61–51 kDa range are presumably serine-proteases, since they can be inhibited by PMSF [14]. The 59–60 kDa serine protease(s) is(are) involved in the PCD process, leading to tracheary element differentiation in *Zinnia*, but not much is known about its activity pattern during different stages of PCD [22,23]. Cysteine proteases inducible by water stress can also fall into this MW range [24]. It should be noted that MC-LR induces the 61–56 and 50–41 kDa alkaline protease activities both at relatively low concentrations, where the first peak of the TUNEL reaction occurs, and as for the TUNEL-positive nuclei, there is a second peak for activity increases. Thus, in the TUNEL reaction, these proteases may indicate the occurrence of PCD (compare Figure 1 and Figure 4d).

The acidic proteases that fell into the 44–28 kDa MW range had decreased activities in the presence of MC-LR (Figure 4). Several acidic protease isoenzymes of this MW range are papain-like cysteine proteases known to be involved in development and cell death [25]. It seems such proteases do not play a role in MC-LR-induced PCD in mustard.

How specific are the effects of MC-LR on nucleases and proteases in *S. alba* seedlings exposed for 4 days? We studied previously the effects of CYN on white mustard under identical conditions [14]. If we compare the effects of these two metabolites, both similarities and differences arise. Under acidic conditions, both toxins induce a slight and transient increase in the 80–75 kDa nuclease isoenzyme activity, while the low MW nucleases (26–20 kDa) seem to be more inducible by CYN as compared to MC-LR. Under non-acidic conditions, the effects of all ssDNase isoenzymes are comparable. Concerning proteases, the activities of 61–51 kDa enzymes show similar changes at treatments with the two cyanotoxins only under alkaline conditions. Under acidic conditions, MC-LR decreases the activities of these isoenzymes, while CYN increases them. The inducibility of the large, 92–260 kDa proteases is more characteristic in the presence of CYN (see Figure 4 and [14] for all protease activities). Thus, in spite of differences, the similar effects of the two cyanotoxins indicate that their PCD-inducing effects are non-specific. That is, these changes in nuclease and protease activities are not directly related to their specific biochemical targets (protein phosphatase and protein synthesis inhibition, respectively).

In conclusion, the occurrence of MC-LR-induced DNA strand breaks, as revealed by TUNEL labeling in mustard seedlings, can be related to an increase in nuclease (e.g., the neutral 26–20 kDa and 80–75 kDA; the acidic ≥100, 55 and 60 kDa isoenzymes) and protease (e.g., the alkalic 61–51 kDa isoenzymes) activities that altogether lead to cell death. Future research will reveal the functioning of these specific hydrolases in the execution of plant PCD. The present research has an important environmental context as well. It may contribute to a better understanding of the toxicity of MC-LR, a cyanobacterial metabolite with a significant impact on aquatic ecosystems and crop plants.

## 4. Materials and Methods

### 4.1. The Purification of MC-LR

MC-LR was purified in our laboratories according to the method of Kós et al. [26] with slight modifications [27]. *Microcystis aeruginosa* BGSD243 cells were collected by centrifugation, then extracted with 80% (*v*/*v*) methanol. After removing methanol, semidry extracts were suspended in Tris-HCl, pH 7.5 (Sigma-Aldrich, St. Louis, MO, USA) and subjected to DE-52 (Whatman, Sigma-Aldrich, St. Louis, MO, USA) ion-exchange chromatography. Fractions containing MC-LR were further purified by HPLC to obtain a MC-LR solution with ≥93% purity.

### 4.2. Plant Material and MC-LR Treatments

White mustard (*Sinapis alba* convar. “Budakalászi sárga”) seeds were surface sterilized and pregerminated, then treated with the cyanotoxin as described [16,28]. MC-LR treatments were conducted under a 14/10 photoperiod at 22 ± 2 °C for 2–8 days, with a focus on the 4-day treatments. MC-LR concentration ranges were set up carefully in preliminary experiments for all cytological and biochemical parameters to detect the most relevant changes induced.

### 4.3. TUNEL Assay

The terminal deoxynucleotidyl transferase mediated dUTP nick end labeling (TUNEL) assay was performed on roots of *S. alba* seedlings treated with MC-LR for 4 days. We followed a method described previously [14] optimized for longitudinal cryosections of plant organs exhibiting high levels of autofluorescence. Therefore, after applying the TUNEL kit (Roche, Basel, Switzerland), we detected positive nuclei with an anti-FITC monoclonal antibody conjugated with alkaline phosphatase (Sigma-Aldrich, St. Louis, MO, USA) followed by bright-field microscopy (Plympus Provis AX-70 microscope, Olympus, Tokyo, Japan). We analyzed the epidermis and inner tissues of young differentiated zones (upper elongation and differentiated zone) of roots and determined the percentage of roots exhibiting high percentage of TUNEL-positive nuclei.

### 4.4. Spectrophotometric and In-Gel Activity Assays of Single-Strand Preferring DNase (SSP Nuclease) Activities

The spectrophotometric assay for SSP nuclease activities was performed according to M-Hamvas et al. [16]. Briefly, crude enzyme extracts were prepared from *S. alba* whole seedlings treated for 2–8 days with Tris-HCl, pH 7.5. The substrate was denatured chicken blood DNA and the medium for enzyme reaction was 15 mM Tris-HCl, pH 6.8 (for the neutral SSP nucleases). Nuclease activity was measured according to the increase of A_260_ measured with a Shimadzu UV-1601 spectrophotometer and expressed as ∆A_260_ h^−1^ mg protein^−1^. Protein content of extracts was measured according to the method of Bradford [29].

The in-gel activity assays for the detection of individual isoenzymes were performed essentially as described by Jámbrik et al. [8,12]; M-Hamvas et al. [14]. Whole *S. alba* seedlings treated for 2–8 days were extracted in a buffer described by Schlereth et al. [30]. 7.5–12.5% gradient polyacrylamide gels of the Laemmli system were used for optimal detection of both high and low MW isoenzymes. These gels contained denatured chicken blood DNA as a substrate for enzymes. Equal amounts (15 μg) of proteins from different samples were loaded onto each well. Protein content of crude enzyme extracts was determined according to Bradford [29]. After the removal of SDS, gels were incubated in either 10 mM Tris-HCl, pH 6.8 (for the neutral SSP nucleases) or 10 mM Tris-maleate, pH 5.5 (for the acidic SSP nucleases). After staining with ethidium bromide (Sigma-Aldrich), enzyme activities were indicated by bands lacking fluorescence. Isoenzyme activities were quantified as relative band intensities calculated with the aid of CPAtlas software.

### 4.5. In-Gel Activity Assays of Protease Activities

Proteins of crude whole-seedling extracts were separated on 10% polyacrylamide gels of the Laemmli system. Equal amounts of proteins (10 μg) from different sample treatments were loaded into the gel wells, as for nuclease activities. The in-gel protease activity assay was performed essentially as described previously [12,14]. Gels contained gelatin as a protease substrate, and after electrophoresis and SDS washout, they were incubated either in 100 mM phosphate buffer, pH 5.0 (for acidic proteases) or 100 mM Tris-HCl, pH 8.0 (for alkaline proteases), then stained with Coomassie Brilliant Blue R250 (Sigma-Aldrich). Protease isoenzymes appeared as clear bands in the blue, Coomasie-stained background. Enzyme activities were quantified as relative band intensities calculated with the aid of CPAtlas software and, for a better visibility of the differences between controls and treatments, were expressed as a percentage of the activities in controls (100%).

### 4.6. Data Analysis

All experiments were repeated at least three times and plotted with the aid of Systat SigmaPlot 10.0 and 12.0 software. Error bars on graphs represent ± SE values. The significance of differences between controls and treatments were evaluated by *t*-tests (TUNEL assay) or after the analysis of variance—One-Way ANOVA by the Tukey/Holm–Sidak method/*t*-tests (enzyme activities) with the aid of Systat Sigma-Plot software; **: significance at *p* < 0.001; *: significance at *p* < 0.05).

## Data Availability

All data supporting reported results are available from the authors’ datasets and can be disclosed if needed.

## Supplementary Materials

The following are available online at https://www.mdpi.com/article/10.3390/plants10102045/s1, Figure S1: The effects of 2 and 4 days of MC-LR treatments of dark-grown *S. alba* seedlings on the in-gel activities of neutral (assayed at pH 6.8) SSP nuclease activities.
