# Supplementary material for: Microcystin-LR, a Cyanobacterial Toxin, Induces DNA Strand Breaks Correlated with Changes in Specific Nuclease and Protease Activities in White Mustard (Sinapis alba) Seedlings
Source: Plants (Basel). 2021 Sep 28;10(10):2045. doi: 10.3390/plants10102045 (PMC8537482; doi:10.3390/plants10102045)
Supplement: Supplementary file 1 [file plants-10-02045-s001.zip › plants-1385158-supplementary.pdf]

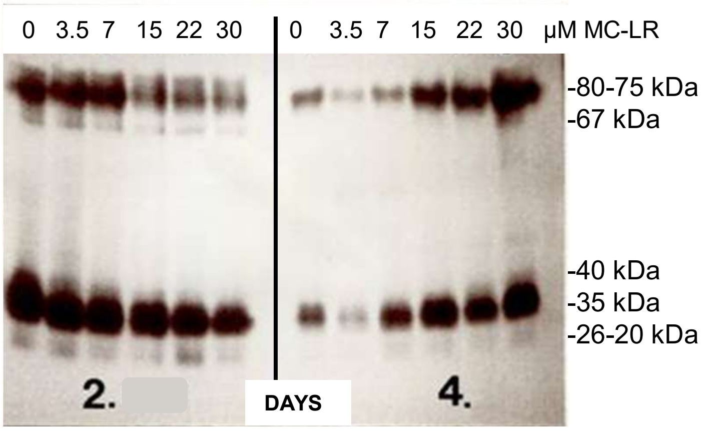

Supplementary Figure S1. The effects of 2 and 4 days of MC-LR treatments of dark-grown *S. alba* seedlings on the in-gel activities of neutral (assayed at pH 6.8) SSP nuclease activities. Treatments of plants were performed under continuous dark condintions. Note that MC-LR decreases after two days and dramatically increases after four days the activities of both the 80-75 kDa and the 40-35 kDa isoenzymes.
